# Hypercapsule is the cornerstone of *Klebsiella pneumoniae* in inducing pyogenic liver abscess

**DOI:** 10.3389/fcimb.2023.1147855

**Published:** 2023-03-31

**Authors:** Dakang Hu, Wenjie Chen, Weiwen Wang, Dongxing Tian, Pan Fu, Ping Ren, Qing Mu, Gang Li, Xiaofei Jiang

**Affiliations:** ^1^ Department of Laboratory Medicine, Huashan Hospital, Fudan University, Shanghai, China; ^2^ Department of Infectious Diseases, Huashan Hospital, Fudan University, Shanghai, China; ^3^ Microbiology Department, Children’s Hospital of Fudan University, Shanghai, China; ^4^ Zhejiang Provincial Demonstration Centre of Laboratory Medicine Experimental Teaching, Wenzhou Medical University, Wenzhou, Zhejiang, China; ^5^ School of Pharmacy, Fudan University, Shanghai, China; ^6^ Department of Laboratory Medicine, Jinshan Hospital of Fudan University, Shanghai, China

**Keywords:** *Klebsiella pneumoniae*, pyogenic liver abscess, mouse lethality test, hypervirulence, mechanism

## Abstract

**Purpose:**

To investigate the mechanisms of *Klebsiella pneumoniae*-induced pyogenic liver abscess (PLA).

**Methods:**

Forty-three *K. pneumoniae* strains from PLAs and 436 from non-PLAs were collected. Their differences were compared for virulence genes and factors, sequence types, and serotypes. Virulence genes *wzi*, *wzy-K1*, and *wzi*+*wzy-K1* were deleted in *K. pneumoniae* NTUH-K2044. Various analyses, such as transmission electron microscopy, neutrophil killing tests, and mouse lethality tests, were used to confirm the consequent changes.

**Results:**

Differences were found between *K. pneumoniae* strains from PLA and non-PLA samples for virulence genes and factors, including metabolism genes (*allS* and *peg-344*), capsular polysaccharide (CPS)-synthesis channel gene (*wzy-K1*), CPS-regulating genes (*p-rmpA*, *p-rmpA2*, and *c-rmpA*), and siderophore genes (*iucA* and *iroN*). When *wzy-K1* was positive, the difference between PLA and non-PLA samples was only observed with *c-rmpA*. Δ*wzi*, Δ*wzy-K1*, and Δ*wzi*Δ*wzy-K1* strains reverted to hypovirulence. In the Kupffer cell stimulation assay, interleukin (IL)-6, IL-12, IL-10, and transforming growth factor-β secretions were found to be equivalent in NTUH-K2044, Δ*wzi*, Δ*wzy-K1*, and Δ*wzi*Δ*wzy-K1* groups. Lower IL-1β and higher tumor necrosis factor-α secretions were observed for Δ*wzi*, Δ*wzy-K1*, and Δ*wzi*Δ*wzy-K1*.

**Conclusions:**

Hypercapsule production is the cornerstone of hypervirulence, regardless of exopolysaccharides. K1 *K. pneumoniae*-induced PLA may decrease core inflammatory cytokines rather than increase anti-inflammatory cytokines. Exopolysaccharides could also attenuate the inflammatory response to aid in the immune escape of *K. pneumoniae*.

## Introduction

Pyogenic liver abscess (PLA) is a common infectious disease in clinical practice (Meddings et al., 2010). PLA was first diagnosed and presented by Ochsner et al. in 1938 and is known to be induced by many kinds of pathogens (Roediger and Lisker-Melman, 2020). The morbidity rate of PLA increased from 11/100,000 to 18/100,000 in Taiwan between 1998 and 2004 (Tsai et al., 2008). In Singapore and Mainland China, 86 – 160 patients suffer from PLA per 100,000 hospitalized patients (Tian et al., 2012; Lo et al., 2015). The mortality rate accounts for 13.0% in patients with PLA (Lo et al., 2015). Therefore, PLA is a fatal disease, whose incidence is increasing and becoming a concern.

Bacteria account for the majority of PLA cases, 85.1% of which are caused by *Klebsiella pneumoniae* and *Escherichia coli* strains with a ratio of almost 6: 1 (Tian et al., 2012; Shi et al., 2017). *K. pneumoniae* exists widely in nature, including healthcare settings (Podschun et al., 2001). In humans, *K. pneumoniae* can colonise various sites, such as the axilla, intestines, and nasopharynx (Lin et al., 2012). *K. pneumoniae* can induce nosocomial infections, such as pneumonia, sepsis, and urinary tract infections; it can also cause community-acquired infections, such as PLA, necrotising fasciitis, endophthalmitis, and meningitis (Russo and Marr, 2019). *K. pneumoniae* can be classified as hypervirulent *K. pneumoniae* (HvKP) and classical *K. pneumoniae* (cKP) (Russo and Marr, 2019). HvKP often possesses extreme resistance to serum and human neutrophils (Gu et al., 2018). *K. pneumoniae* strains causing PLA are mostly hypervirulent, particularly those typed as K1 and K2 (Qu et al., 2015). However, to our knowledge, the mechanisms of development of *K. pneumoniae*-induced PLA are currently unknown.

*K. pneumoniae* can harbour multiple virulence factors, such as exopolysaccharide (EPS), capsular polysaccharide (CPS), lipopolysaccharide, siderophores, fimbriae, allantoin metabolism, outer membrane proteins, and porins (Paczosa and Mecsas, 2016). These factors contribute to the virulence of *K. pneumoniae*, such as the anti-phagocytosis, anti-complement, and anti-biofilm effects of CPS, and are encoded by a series of virulence genes. For instance, CPS synthesis is controlled by the *cps* cluster and is regulated by the regulator of mucoid phenotype A gene (*rmpA*). In addition, CPS anchoring is dependent on the gene *wzi* (Paczosa and Mecsas, 2016). Therefore, *K. pneumoniae* virulence has considerable complexity, which needs to be studied further.

The purpose of the present study was to analyse important virulence factors in *K. pneumoniae* by comparing gene positivity in PLA-inducing and non-PLA-inducing strains and by determining the effect of deletions of specific genes on *K. pneumoniae* virulence, focusing on genes related to CPS and EPS.

## Materials and methods

### K. pneumoniae strains

In this study, 43 and 436 K*. pneumoniae* strains were obtained from PLA and non-PLA patients, respectively. All the strains were isolated from distinct patients. The PLA strains were isolated from abscess, drainage, and puncture fluid specimens at the Department of Infectious Diseases, the First Affiliated Hospital of Zhejiang University in 2017. PLA was diagnosed based on pathogen and imaging evidences (B-mode ultrasonography and computed X-ray tomography). The non-PLA strains were obtained from seven hospitals in China between January 2017 and February 2018: Huashan Hospital, 180 strains; Jinshan Hospital, 28 strains; Taizhou Municipal Hospital, 84 strains; the First Affiliated Hospital of Guangxi Medical University, 20 strains; Kunming Yan’an Hospital, 34 strains; Sixth Hospital of Shanxi Medical University, 60 strains; Shandong Provincial Hospital Affiliated with Shandong University, 30 strains. Their sources included sputum (225, 58.5%), urine (98, 22.5%), blood (29, 6.7%), and others (54, 12.4%).

The specimens were stored at -80°C until use. Sheep blood agar plates were used to culture the strains, followed by identification using matrix-assisted laser desorption/ionization time-of-flight mass spectrometry (Bruker Daltonics Inc., Fremont, CA, USA). The standard strains *Pseudomonas aeruginosa* ATCC 27853, *K. pneumoniae* ATCC 700603, and *E. coli* ATCC 25922 were used as controls.

*K. pneumoniae* NTUH-K2044 (Accession number: AP006725.1), a typical HvKP, was originally isolated from the Department of Internal Medicine, National Taiwan University Hospital, Taipei, Taiwan. *K. pneumoniae* HS11286 (Accession number: CP003200.1), a K47 strain expressing *bla*_KPC_ and showing hypovirulence, was originally isolated from the Department of Laboratory Medicine, Huashan Hospital, Fudan University, Shanghai, China. Strains NTUH-K2044 and HS11286 were used as controls for strain morphological tests, string tests, capsule staining, periodic acid–Schiff staining, fitness analyses, quantitative PCR, mouse lethality tests, and *Galleria mellonella* lethality tests. Strain NTUH-K2044 was also a target for gene deletions (*wzi*, *wzy-K1*, and *wzi*+*wzy-K1*). The genetic traits of NTUH-K2044 and HS11286 were shown in **Table 1**.

**Table 1:** Genetic backgrounds of NTUH-K2044 and HS11286.

Strain	ST	Serotype	*allS*	*peg-344*	*wzy-K1*	*entB*	*irp2*	*iroN*	*iucA*	*fimH*	*mrkD*	*c-rmpA*	*p-rmpA*	*p-rmpA2*	*wzi*
NTUH-K2044	23	K1	+	+	+	+	+	+	+	+	+	+	+	+	+
HS11286	11	K47	–	–	–	+	+	–	–	+	+	–	–	–	+

ST, sequence type; +, positive; -, negative.

The study was a retrospective investigation; thus, approval to use the 43 and 436 K*. pneumoniae* strains was waived.

### Multilocus sequence typing

The primers of seven housekeeping genes (*gapA, infB, mdh, pgi, phoE, rpoB*, and *tonB*) are shown in **Table S1**. The QIAamp DNA mini kit (catalogue number: 51304, QIAGEN, Düsseldorf, Germany) was used to extract DNA from the *K. pneumoniae* strains following the manufacturer’s protocol. PCR was used to amplify the housekeeping genes, and the products were sequenced using an ABI 3730XL DNA Analyzer (Applied Biosystems, San Ramon, CA, USA). Sequence types (STs) were obtained after searching the *K. pneumoniae* MLST database (http://bigsdb.pasteur.fr/cgi-bin/bigsdb/bigsdb.pl?db=pubmlst_klebsiella_seqdef&page=sequenceQuery).

### Determination of serotypes and virulence genes

The *wzi* locus was sequenced to determine serotypes of the *K. pneumoniae* strains by searching the Institute Pasteur database (http://bigsdb.pasteur.fr/cgi-bin/bigsdb/bigsdb.pl?db=pubmlst_klebsiella_seqdef&page=sequenceQuery).

Virulence genes, *wzy-K1*, *allS*, *entB*, *irp2*, *iroN*, *iucA*, *fimH*, *mrkD*, *p-rmpA*, *p-rmpA2*, *c-rmpA*, *peg-344*, and *wzi* (Compain et al., 2014; Gu et al., 2018; Russo et al., 2018), were analysed using a Veriti PCR system (Applied Biosystems). The primers used are shown in **Table S1**. NTUH-K2044 was used as the positive control. In the subsequent agarose electrophoresis, a proper band in line with the control is regarded as a positive gene.

### Gene deletions in *K. pneumoniae* NTUH-K2044

Deletions of *wzi*+*wzy-K1*, and *wzi*+*wzy-K1* were constructed using the lambda Red recombination method as previously described (Datsenko and Wanner, 2000). The primers used are shown in **Table S1**.

### String test

String tests were performed thrice per strain as previously described (Shon et al., 2013). The test was considered positive when the produced string was longer than five mm.

### Capsule staining

*K. pneumoniae* strains were stained to distinguish their hypercapsules according to the manufacturer’s instructions (catalogue number: BA-4039; BASO, Zhuhai, China).

### Periodic acid–Schiff staining

Periodic acid–Schiff staining was performed to detect EPS according to the manufacturer’s protocol (catalogue number: BA4080A; BASO, Zhuhai, China).

### Transmission electron microscopy

TEM was performed using a Tecnai G2 Spirit Twin Electron Microscope (FEI, Hillsboro, USA), as previously reported (Ernst et al., 2020). *K. pneumoniae* strains were cultured overnight on sheep blood agar plates. Luria Bertani (LB) broth was then used to culture them to mid-log phase. The strains were collected after centrifugation at 11000 g for 5 min. The sediments were immersed at 2.5% glutaraldehyde overnight, followed by washing thrice with phosphate buffered saline (PBS) and fixation with 1% osmic acid for 1.5 h. The pellets were then washed thrice with PBS, followed by dehydration with grades of alcohol (30%, 50%, 70%, 80%, 95%: 15 min; 100%: 2 × 10 min). Infiltration and embedding were performed using acetone and Epon-812 respectively, followed by polymerization at 60°C for 48 h. The pellets were sliced and stained by 2% uranyl acetate water (10 min) and lead citrate (10 min) before TEM.

### Human neutrophil killing assay

This protocol is based on a previously reported method (Deleo et al., 2014). The human neutrophil killing assay was approved by the Ethics Committee of Huashan Hospital (Shanghai, China). Neutrophils (1 × 10^6^) from healthy volunteers and 1 × 10^6^ colony forming unit (CFU) of opsonized *K. pneumoniae* were mixed in RPMI/H medium at 37°C for zero and 60 min with gentle rotation. One percent saponin was added to each tube, mixed, and chilled on ice for 15 min. The mixture was diluted and cultured overnight on LB agar plates. Viable colonies were counted, and the survival ratios were calculated relative to the zero min point. Each strain was tested three times.

### Serum killing assay

This protocol is based on a previously reported method (Lin et al., 2014). The serum killing assay was approved by the Ethics Committee of Huashan Hospital (Shanghai, China). Human blood was collected from ten healthy volunteers, and sera were isolated. The sera were mixed, divided into 500 μL aliquots, and stored at -80°C prior to use. Mid-log-phase *K. pneumoniae* strains were washed twice with normal saline and readjusted to 4 × 10^6^ CFU/mL, 25 μL of which were added to 75 μL of pooled sera in a 12-well plate (Corning Incorporated, Corning, NY). After zero, one, two, and three h of incubation at 37°C, the mixture was diluted and cultured overnight on LB agar plates. Viable colonies were counted, and the survival ratio was calculated relative to the zero h point. Each strain was tested three times.

### Fitness analysis

Fitness of the *K. pneumoniae* strains was determined as previously described (Liu et al., 2016). Overnight cultures were diluted to an optical density at 600 nm (OD_600_) of 0.001 and incubated at 37°C under aerobic conditions, monitoring the OD_600_ every 30 min on a microplate reader (BioTek Synergy H1, Winooski, VT, USA).

### Real-time quantitative PCR

Real-time quantitative PCR on an Applied Biosystems 7500 System was used to evaluate the expression of *p-rmpA*, *p-rmpA2*, *c-rmpA, manC*, and *galF*, with 16S rRNA as the reference gene. The primers used are shown in **Table S1**. The analyses were performed according to the manufacturer’s protocol of the SYBR Green qPCR Mix (catalogue number: FS-Q1002; FOREVER STAR, Beijing, China).

### Mouse lethality test

Six-week-old pathogen-free female BALB/c mice (four per group) were inoculated intraperitoneally with 100 μL of the *K. pneumoniae* strains (10^2^–10^7^ CFU, mid-logarithmic growth) that had been washed twice with normal saline (Mizuta et al., 1983). The mice were then observed for 14 days. Lethality dose 50 (LD_50_) values and survival curves were obtained as reported in a previous study (Reed and Muench, 1938).

### Kupffer cell stimulation assay

The Kupffer cells were isolated from specific pathogen-free mice and the cell stimulation assays were performed as described previously (Lalitha et al., 2017; Hoh et al., 2019). Six hundred microlitres of 1 × 10^4^ Kupffer cells were inoculated into each well of a 48-well plate, followed by the addition of 2.0 μL of lipopolysaccharide (1.5 mg/mL) and interferon (0.25 mg/mL). After incubation at 37°C in a 5.0% CO_2_ atmosphere for two h, 20 μL of a *K. pneumoniae* strain (5 × 10^6^ CFU/mL) were added, resulting in a multiplicity of infection of 10. After four, eight, and twelve h of further incubation, the suspensions were collected and centrifuged at 10621g for ten min. Interleukin (IL)-1β, IL-6, IL-10, IL-12, tumor necrosis factor-α (TNF-α), and transforming growth factor-β (TGF-β) were measured in the supernatants as indicated in ELISAs.

### ELISA

ELISAs were used to detect IL-1β, IL-6, IL-10, IL-12, TNF-α, and TGF-β, according to the manufacturer’s instructions (catalogue numbers: F10770, F10830, F10870, F10880, F11630, and F11591; Shanghai Westang Biotech Inc., Shanghai, China).

### Statistical analysis

GraphPad Prism 8 software (GraphPad Software Inc., San Diego, CA, USA) was used for Chi-square tests, Fisher’s exact tests, one-way ANOVA, factorial design analysis of variance, and Kruskal–Wallis tests between groups. Significance was considered at *p* < 0.05.

## Results

### Significant differences between *K. pneumoniae* from PLA and non-PLA

**Figure 1:**
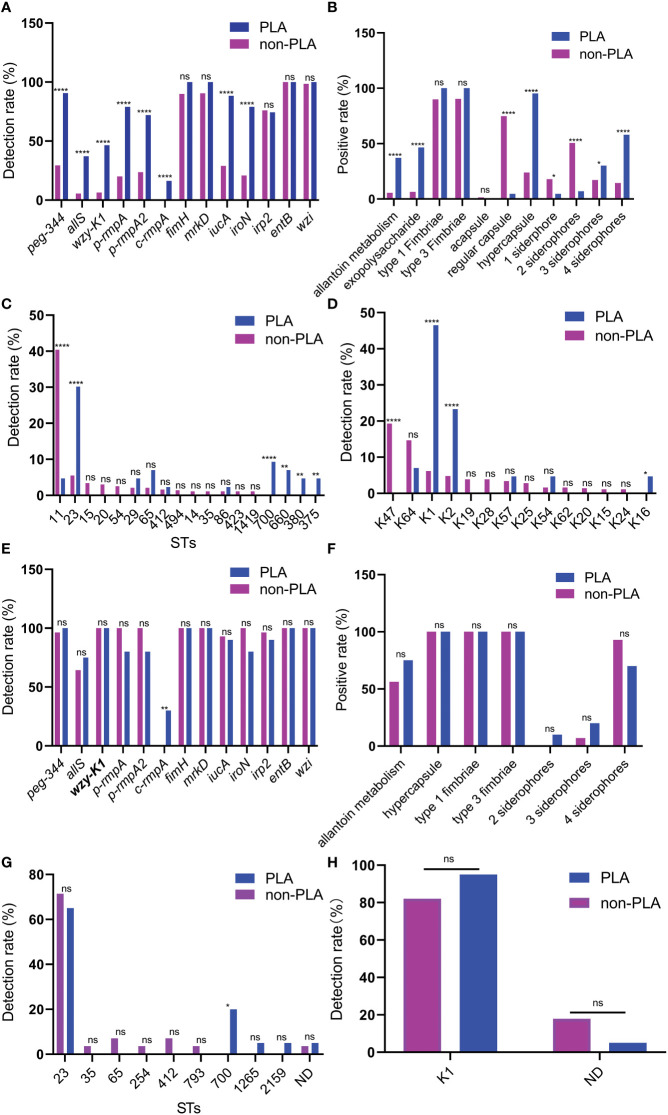
Differences between *Klebsiella pneumoniae* from PLA and non-PLA samples. **(A)** Differences in virulence genes; **(B)** Differences in virulence factors; **(C)** Differences in STs; **(D)** Differences in serotypes; **(E)** Differences in virulence genes when *wzy-K1* is positive; **(F)** Differences in virulence factors when *wzy-K1* is positive; **(G)** Differences in STs when *wzy-K1* is positive; **(H)** Differences in serotypes when *wzy-K1* is positive. PLA: pyogenic liver abscess; PLA group: n = 43 **(A–D)** n = 20 **(E–H)** non-PLA group: n = 436 **(A–D)** n = 28 **(E–H)** ns: not significant; *p < 0.05; **p < 0.01; ****p < 0.0001; ST, sequence type; ND, not defined. Chi-square tests and Fisher’s exact tests were used to compare the positive rates between PLA and non-PLA groups.

As shown in (**Figures 1A, B**), higher rates were found in *K. pneumoniae* strains from PLA than those from non-PLA for virulence genes and factors, including metabolism (*allS* and *peg-344*), CPS-synthesis channel (*wzy-K1*), CPS-regulating (*p-rmpA*, *p-rmpA2*, and *c-rmpA*), and siderophore (*iucA* and *iroN*) genes. For STs, a lower rate of ST11 and higher rates of ST23/ST700/ST660/ST380/ST375 were observed in strains from PLA; for serotypes, a lower rate of K47 and higher rates of K1/K2/K16 were found in strains from PLA (**Figures 1C, D**).

Since virulence gene *wzy-K1* was with significant difference (*p* < 0.0001) and nearly half-positive (20/43) in strains from PLA, it was used for stratification analysis. When *wzy-K1* was positive, significant differences were observed only with *c-rmpA* (*p* < 0.01) and ST700 (*p* < 0.05) (**Figures 1E, G**): higher rates of *c-rmpA* (6/20) and ST700 (4/20) in strains from PLA; no significant differences were found for virulence factors and serotypes (**Figures 1F, H**). When *wzy-K1* was positive, the positivity of virulence genes increased, except for *fimH*, *mrkD*, *irp2*, *entB*, and *wzi* (**Figures 1A, E**).

### Roles of CPS and EPS in PLA

**Figure 2:**
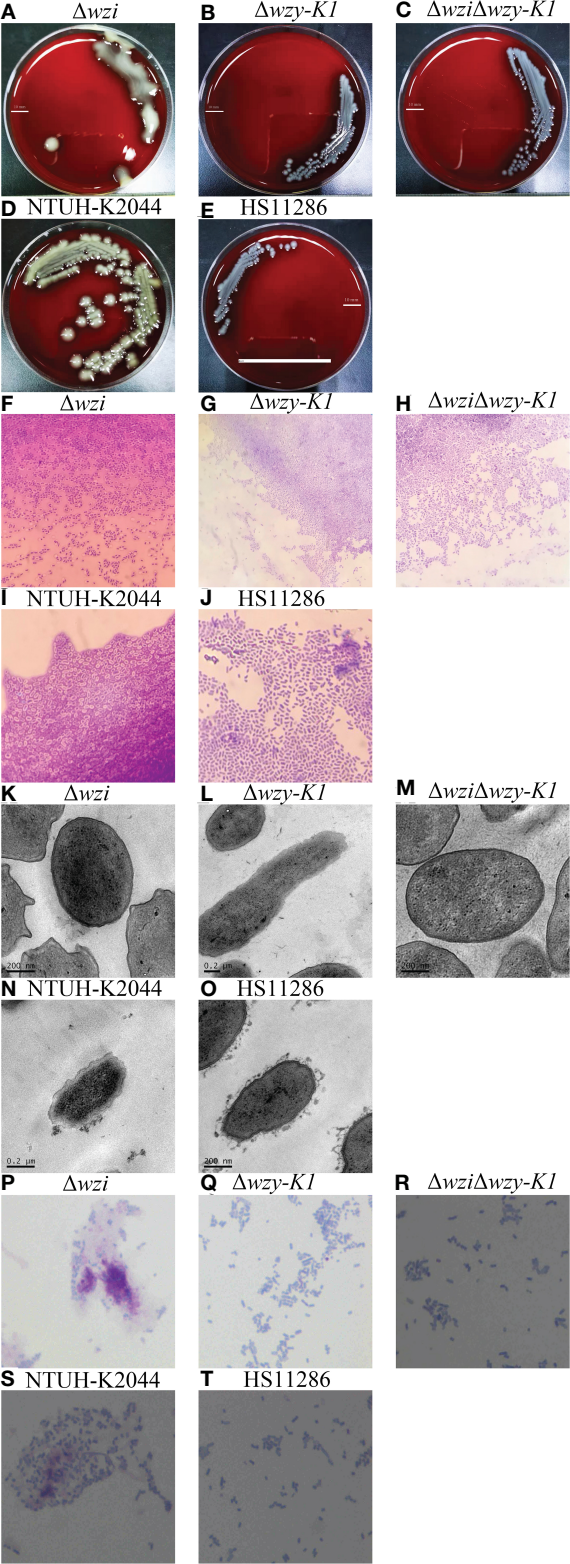
Morphological effects of deletions of *wzi*, *wzy-K1*, and *wzi+wzy-K1* on *Klebsiella pneumoniae*. **(A–E)** Colonies of Δ*wzi*, Δ*wzy-K1*, Δ*wzi*Δ*wzy-K1*, NTUH-K2044, and HS11286; **(F–J)** Capsule staining of Δ*wzi*, Δ*wzy-K1*, Δ*wzi*Δ*wzy-K1*, NTUH-K2044, and HS11286; **(K–O)** Transmission electron microscopy of Δ*wzi*, Δ*wzy-K1*, Δ*wzi*Δ*wzy-K1*, NTUH-K2044, and HS11286; **(P–T)** Periodic acid-Schiff staining of Δ*wzi*, Δ*wzy-K1*, Δ*wzi*Δ*wzy-K1*, NTUH-K2044, and HS11286. The lines in **(A–E)** represent 10 mm; *K. pneumoniae* strains are purple and rod-shaped, and their transparent surroundings are hypercapsules (×1000) **(F–J)**. The prominent “black edge” on the edge of the cell is the cell wall, and the loose material outside is the capsule **(K–O)**. *K. pneumoniae* strains are blue and rod-shaped; the red fluffy masses are exopolysaccharides **(P–T)**. The diameters of Δ*wzi*, Δ*wzy-K1*, Δ*wzi*Δ*wzy-K1*, NTUH-K2044, and HS11286 fell within such ranges: 8.0-10.0, 6.0-8.0, 2.0-4.0, 2.0-4.0, and 2.0-4.0 mm **(A–E)**.

To investigate the roles of CPS and EPS in PLA, *wzi*, *wzy-K1*, and *wzi*+*wzy-K1* were deleted in NTUH-K2044. The colonies were larger with Δ*wzi* and smaller with Δ*wzy-K1* and Δ*wzi*Δ*wzy-K1* than with NTUH-K2044 (**Figures 2A–E**). The string test was positive for Δ*wzi* but negative for Δ*wzy-K1* and Δ*wzi*Δ*wzy-K1*. Capsule staining showed that Δ*wzi*, Δ*wzy-K1*, and Δ*wzi*Δ*wzy-K1* did not have hypercapsules (**Figures 2F–J**). TEM suggested that Δ*wzi* and Δ*wzi*Δ*wzy-K1* were without a capsule, while Δ*wzy-K1* had a thin capsule (**Figures 2K–O**). Periodic acid–Schiff staining confirmed the presence of EPS in Δwzi and NTUH-K2044 and its abscence in Δ*wzy-K1*, Δ*wzi*Δ*wzy-K1*, and HS11286 (**Figures 2P–T**).

**Figure 3:**
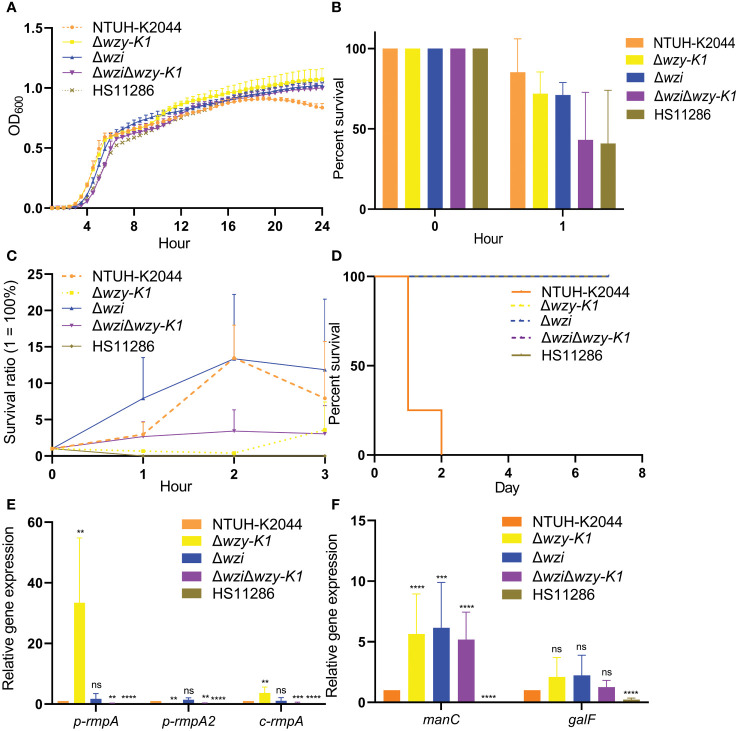
Non-morphological effects of deletions of *wzi*, *wzy-K1*, and *wzi+wzy-K1* on *Klebsiella pneumoniae*. **(A)** Growth curves; **(B)** Survival percentages in human neutrophil killing assay; **(C)** Survival ratios in serum killing assay; **(D)** Survival curves of mice infected with knockouts; **(E)** Relative expression of *p-rmpA*, *p-rmpA2*, and *c-rmpA* in knockouts; **(F)** Relative expression of *manC* and *galF* in knockouts. OD_600_: optical density at 600 nm; ns: not significant; **p < 0.01; ***p < 0.001; ****p < 0.0001. NTUH-K2044 and HS11286 were used as positive and negative controls, respectively. One-way ANOVA showed no significant differences among the 5 groups **(A)** (F = 0.0455, *p* = 0.9871). The values of means and standard deviations were 85.20/20.83, 71.90/13.53, 71.07/7.73, 43.17/29.60, and 40.83/33.18 respectively for NTUH-K2044, Δ*wzy-K1*, Δ*wzi*, Δ*wzi*Δ*wzy-K1*, and HS11286 after 1-h incubation **(B)**. Homogeneity test of variance showed *p* = 0.0005 using Levene’s test. Kruskal-Wallis test presented a *p* value of 0.0065 among the 5 groups; while HS11286 was omitted, *p* = 0.1169 among the other 4 groups **(C)**. The LD_50_ value of 10^6^ CFU were for NTUH-K2044 in mouse lethality test, of which was >10^7^ CFU for other 4 groups. The inoculation of 10^6^ CFU was performed for the survival curve. Log-rank (Mantel-Cox) test showed χ2 = 23.0252 and *p* = 0.0001 among the 5 groups. Except NTUH-K2044, the other 4 groups showed the same survival curve; their comparison showed χ2 = 7.6037, *p* = 0.0058 **(D)**. The relative expressions of *p-rmpA*, *p-rmpA2*, and *c-rmpA* were compared with those in NTUH-K2044. For the expression of *p-rmpA*, the means and standard deviations of △CT values were 19.52/0.87, 14.81/1.38, 19.13/1.07, 22.10/0.73, and 27.62/2.53 respectively in NTUH-K2044, Δ*wzy-K1*, Δ*wzi*, Δ*wzi*Δ*wzy-K1*, and HS11286; for the expression of *p-rmpA2*, such values were 16.03/0.82, 25.88/2.33, 15.72/1.49, 19.92/2.49, and 25.18/2.36; for the expression of *c-rmpA*, such values were 16.83/1.80, 15.19/2.59, 17.31/2.56, 19.29/1.38, and 26.51/1.76 **(E)**. The relative expressions of *manC* and *galF* were compared with those in NTUH-K2044. For the expression of *manC*, the means and standard deviations of △CT values were 18.62/0.95, 30.47/1.86, 16.89/1.88, 16.58/0.60, and 27.76/1.26 respectively in NTUH-K2044, Δ*wzy-K1*, Δ*wzi*, Δ*wzi*Δ*wzy-K1*, and HS11286; for the expression of *galF*, such values were 13.06/1.05, 16.79/0.69, 12.51/0.78, 13.33/0.81, and 15.36/0.88 **(F)**.

Growth curves showed no significant differences among NTUH-K2044, Δ*wzi*, Δ*wzy-K1*, Δ*wzi*Δ*wzy-K1*, and HS11286 (**Figure 3A**). The survival percentages were > 70.0% for Δ*wzi* and Δ*wzy-K1* in the human neutrophil killing assay, while they decreased to ~40.0% for Δ*wzi*Δ*wzy-K1* and HS11286 (**Figure 3B**). No significant differences were found among NTUH-K2044, Δ*wzi*, Δ*wzy-K1*, and Δ*wzi*Δ*wzy-K1* for serum resistance (**Figure 3C**). Δ*wzi*, Δ*wzy-K1*, and Δ*wzi*Δ*wzy-K1* all lost hypervirulence in the mouse lethality test (**Figure 3D**).

**Figure 3E** shows lower expression of *p-rmpA, p-rmpA2*, and *c-rmpA* in Δ*wzi*, Δ*wzy-K1*, and Δ*wzi*Δ*wzy-K1* than in NTUH-K2044. **Figure 3F** shows equal expression of *galF* and higher expression of *manC* in Δ*wzi*, Δ*wzy-K1*, and Δ*wzi*Δ*wzy-K1* than in NTUH-K2044.

### Impact of CPS and EPS on cytokine secretion

**Figure 4:**
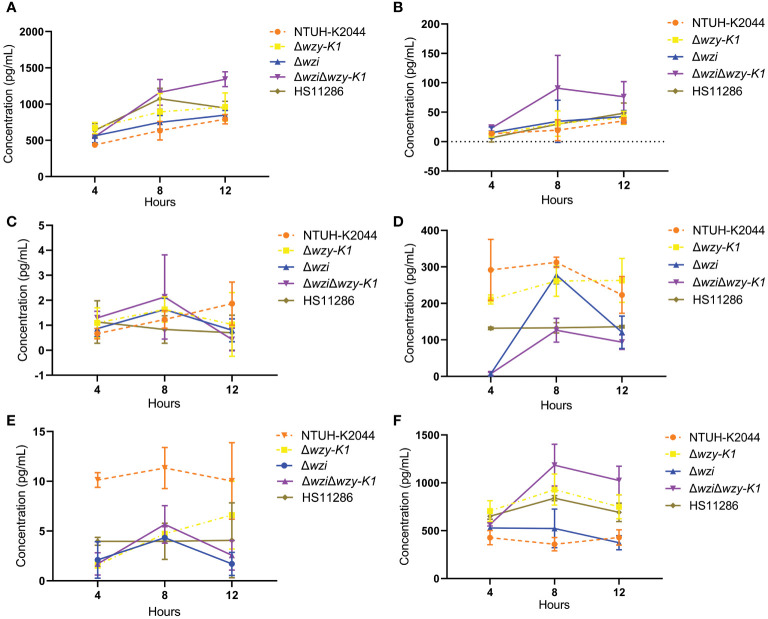
Effects of deletions of *wzi*, *wzy-K1*, and *wzi+wzy-K1* in *Klebsiella pneumoniae* on cytokine secretion by Kupffer cells. **(A)** IL-6 secretion; **(B)** IL-12 secretion; **(C)** IL-10 secretion; **(D)** TGF-β secretion; **(E)** IL-1β secretion; **(F)** TNF-α secretion. IL: interleukin; TGF-β: transforming growth factor-beta; TNF-α: tumor necrosis factor-alpha. NTUH-K2044 and HS11286 were used as positive and negative controls, respectively. Homogeneity test of variance showed *p* = 0.0106 using Levene’s test. Kruskal-Wallis test presented a *p* value of 0.4576 among the 5 groups **(A)**. The factorial design analysis of variance showed significant differences among the 5 groups: F = 9.7138, *p* = 0.0018; further one-one comparisons confirmed all *p* values > 0.05; Comparison of each time point suggested F = 9.0737 and *p* = 0.0092. Analysis of interaction between groups and time points showed F = 0.8345, *p* = 0.5834 **(B)**. ANOVA showed no significant differences among the 5 groups: F = 0.7462, *p* = 0.5823; Analysis of interaction between groups and time points showed F = 1.1687, *p* = 0.3644 **(C)**. Normality test showed *p* = 0.0192 using Kolmogorov-Smirnov test. Kruskal-Wallis test presented a *p* value of 0.0264 among the 5 groups; further one-one comparisons confirmed all *p* values > 0.05 **(D)**. ANOVA showed significant differences among the 5 groups: F = 38.9224, *p* < 0.0001 with NTUH-K2044 being higher than all the other 4 groups; the latter 4 groups all equalled: *p* > 0.05. Analysis of interaction between groups and time points showed F = 1.1674, *p* = 0.3651 **(E)**. ANOVA showed significant differences among the 5 groups: F = 36.4485, *p* < 0.0001; further one-one comparisons confirmed such comparisons with *p* values > 0.05: NTUH-K2044 vs. Δ*wzi*、Δ*wzy-K1* vs. Δ*wzi*Δ*wzy-K1*、Δ*wzy-K1* vs. HS11286、Δ*wzi*Δ*wzy-K1* vs. HS11286, the others being with *p* < 0.05. Analysis of interaction between groups and time points showed F = 4.8494, *p* = 0.0020 **(F)**.

No significant differences were found among Δ*wzi*, Δ*wzy-K1*, Δ*wzi*Δ*wzy-K1*, and NTUH-K2044 for IL-6, IL-12, IL-10, and TGF-β secretions (**Figures 4A–D**) in the Kupffer cell stimulation assay. The secretion of IL-1β was lower for Δ*wzi*, Δ*wzy-K1*, and Δ*wzi*Δ*wzy-K1* than for NTUH-K2044 (**Figure 4E**), which was the opposite of that observed with TNF-α (**Figure 4F**).

## Discussion

This investigation showed the differences between *K. pneumoniae* strains isolated from PLA and non-PLA samples; the roles of CPS and EPS in PLA; and the effects of CPS and EPS on cytokine secretion. Mutants of NTUH-K2044 were constructed through the deletions of *wzy-K1*, *wzi*, and the both. The gene *wzy-K1* is a key virulence gene in K1 *K. pneumoniae*, encoding the Wzx/Wzy channel that is crucial for the synthesis of CPS and EPS (Schmid et al., 2015). The gene *wzi* encodes Wzi, which is the only anchor for CPS (Paczosa and Mecsas, 2016).

**Figure 1** shows the differences between *K. pneumoniae* strains isolated from PLA and non-PLA samples. PLA is commonly community-acquired while a high proportion of non-PLA is hospital-acquired; **Figures 1C**, **D** confirmed such differences: ST23/ST65/ST700/ST660 and K1/K2 were widely found in *K. pneumoniae* strains from PLA while ST11 and K47 accounted for the majority in those from non-PLA. The proportion of serotype K1 in *K. pneumoniae* from PLA declined to 46.5% (20/43) compared with that in a previous report from five to nine years ago (68.9%, 31/45) (χ^2^ = 4.5186, *p* = 0.0335) (Qu et al., 2015), while the proportion of K2 was equal (9/45 vs. 10/43) (χ^2^ = 0.1377, *p* = 0.7106). The proportion of ST23 also declined from 57.8% (26/45) to 30.2% (13/43) (χ^2^ = 6.761, *p* = 0.0093). Nevertheless, K1 still dominates the serotypes of *K. pneumoniae* strains from PLA. Another study (Yu et al., 2006) showed that *wzy-K1* could confer hypercapsule production in the absence of *rmpA* and *rmpA2*. Nearly half of the *K. pneumoniae* strains from PLA harboured *wzy-K1*, suggesting its important role in PLA. Based on positive *wzy-K1*, gene *c-rmpA* was the only difference between *K. pneumoniae* from PLA and non-PLA samples, which indicates a possible role of *c-rmpA* in PLA. The mere existence of *c-rmpA* in strains from PLA suggests its high specificity to PLA. Serotypes of *K. pneumoniae* strains could be determined by such methods: serological test and regular PCR to detect *wzy-K1*、PCR amplification and sequencing of *wzi* (Fang et al., 2007; Yeh et al., 2007; Turton et al., 2010). Usually, the results of such three methods show extremely high consistency. Gene *wzy-K1* is thought to be in line with K1 *K. pneumoniae* (Struve et al., 2005; Yeh et al., 2006; Fang et al., 2010). **Figure 1H** presented some inconsistency. In this study, the last method was used to define serotypes. Presumably, mutations of *wzi* caused the inconsistency.

**Figures 2F, K** confirm the disappearance of the capsule in Δ*wzi*. Compared to NTUH-K2044, the larger colonies of Δ*wzi* (**Figure 2A**) resulted from the continuous synthesis of EPS and ropy CPS. **Figures 2G**, **2L**, and **2Q** verified the thin capsule and disappearance of EPS in Δ*wzy-K1*, which resulted in smaller colonies (**Figure 2B**) compared to those of NTUH-K2044. The disappearance of both CPS and EPS was confirmed in Δ*wzi*Δ*wzy-K1* (**Figures 2H, M, R**), which also resulted in smaller colonies (**Figure 2C**). NTUH-K2044 harbours 4 siderophores, i.e. aerobactin, salmochelin, yersiniabactin, and enterobactin. All three deletions showed no fitness cost (**Figure 3A**) and the four siderophores were retained. Virulence genes *manC* and *galF* are located at the downstream and upstream of *cps* cluster, which are often used to indicate the synthesis of CPS (Palacios et al., 2018; Peng et al., 2018). The higher expression of *p-rmpA*, *c-rmpA*, and *manC* in Δ*wzy-K1* resulted from the continuous synthesis of CPS precursors and blocking of the Wzx/Wzy channel (**Figures 3E, F**). With a functional Wzx/Wzy channel, normal expression of *rmpAs* was found in Δ*wzi*; Δ*wzi*Δ*wzy-K1* resulted in decreased expression of *rmpAs*. No significant impact of serum resistance was found in Δ*wzi*, Δ*wzy-K1*, and Δ*wzi*Δ*wzy-K1* (**Figure 3C**). The survival rates were > 70.0% for Δ*wzi* and Δ*wzy-K1* in the human neutrophil killing assay, while it was reduced to ~40.0% for Δ*wzi*Δ*wzy-K1* (**Figure 3B**), indicating the role of EPS and thin capsules in protection against neutrophils. **Figure 3D** confirms the hypovirulence of Δ*wzi*, Δ*wzy-K1*, and Δ*wzi*Δ*wzy-K1*, although they all possessed four siderophores, suggesting that the hypercapsule is the core factor of hypervirulence rather than EPS.

Kupffer cells in the liver can secrete large amounts of proinflammatory cytokines, such as TNF-α, IL-1β, IL-6, and IL-12, and chemokines, such as C-X-C motif chemokine ligand 1 (CXCL1), CXCL2, CXCL3, CXCL8, and CXCL4 when exposed to bacteria (Liaskou et al., 2012). However, many anti-inflammatory cytokines are synthesised, such as IL-10 and TGF-β, under normal conditions. **Figure 4** shows differences in IL-1β and TNF-α, but not IL-6, IL-12, IL-10, or TGF-β secretion in Δ*wzi*, Δ*wzy-K1*, and Δ*wzi*Δ*wzy-K1* versus NTUH-K2044. IL-1β decreased with all three deletions (**Figure 4E**). The trend for TNF-α was not the same versus NTUH-K2044, increasing with Δ*wzy-K1* and Δ*wzi*Δ*wzy-K1* but equal with Δ*wzi* (**Figure 4F**). **Figure 4** indicates that EPS in NTUH-K2044 contributes to immune escape from macrophages and attenuates the immune response of Kupffer cells, which is consistent with previous reports (Yoshida et al., 2000; Yoshida et al., 2001). The decreased IL-1β production of Δ*wzi*, Δ*wzy-K1*, and Δ*wzi*Δ*wzy-K1*, equal to that of HS11286 (**Figure 4E**), reflects the roles of CPS and EPS in HvKP, inducing the secretion of IL-1β from Kupffer cells and consequent hepatic lesions. The manifestations of TNF-α and IL-1β are likely to be contradictory. However, TNF-α plays a more important role in the process of PLA (Li et al., 2014). HS11286 harbours yersiniabactin and enterobactin according to **Table 1**. Therefore, Δ*wzy-K1* shows traits similar to those of HS11286: thin capsule, no EPS, and types 1 and 3 fimbriae. The crucial difference between Δ*wzy-K1* and HS11286 is the numbers of siderophores: four vs. two. **Figure 4** confirms equal secretion of the six cytokines between Δ*wzy-K1* and HS11286, which suggests the inability of excessive siderophores to induce inflammatory responses in Kupffer cells.

This study has some limitations. First, the sample size of strains from PLA is small, which may bring bias in **Figure 1**. Second, the PLA mouse model is difficult to establish because of the hypovirulence of Δ*wzi*, Δ*wzy-K1*, and Δ*wzi*Δ*wzy-K1*. The majority of methods was then conducted *in vitro* in our study.

## Conclusions

In summary, the presence of a hypercapsule is the cornerstone of hypervirulence regardless of the presence of EPS. The strategy of K1 *K. pneumoniae* in inducing PLA is to decrease the expression of core inflammatory cytokines rather than increase expression of anti-inflammatory cytokines. In contrast with CPS, EPS can effectively reduce the inflammatory response to aid in the immune escape of *K. pneumoniae*.

## Data availability statement

The datasets presented in this study can be found in online repositories. The names of the repository/repositories and accession number(s) can be found below: https://pan.baidu.com/s/1iL6WDemsLyPJepNNrYbTiQ?pwd=1234; Key: 1234.

## Ethics statement

The studies involving human participants were reviewed and approved by the Ethics Committee of Huashan Hospital (Shanghai, China) (ethical approval No. 2021-484). Written informed consent for participation was not required for this study in accordance with the national legislation and the institutional requirements. The animal study was reviewed and approved by the Institutional Animal Care and Use Committee of the School of Pharmacy, Fudan University (Ethical approval No. 201603-TY-MQ-01).

## Author contributions

DH, WC, and WW conceived the study. GL, and XJ collected and identified the strains. DH, WC, WW, PF, and DT performed PCR and MLST analyses, string tests, capsular staining, periodic acid–Schiff staining, ELISA, gene deletion, Galleria mellonella lethality and fitness tests. PR and QM performed mouse lethality tests. DH, WC, and WW wrote the manuscript which was revised by XJ and GL. All authors contributed to the article and approved the submitted version.
