# Supplementary material for: Hypercapsule is the cornerstone of Klebsiella pneumoniae in inducing pyogenic liver abscess
Source: Front Cell Infect Microbiol. 2023 Mar 31;13:1147855. doi: 10.3389/fcimb.2023.1147855 (PMC10102340; doi:10.3389/fcimb.2023.1147855)
Supplement: Supplementary file 1 [file Table_1.docx]

**Hypercapsule is the cornerstone of *Klebsiella pneumoniae* in inducing pyogenic liver abscess**

Dakang Hu^1,†^, Wenjie Chen^2, †^, Weiwen Wang^1, †^, Dongxing Tian^1^, Pan Fu^3,1^, Ping Ren^4^, Qing Mu^5^, Gang Li^6,*^, Xiaofei Jiang^1,*^

^1^Department of Laboratory Medicine, Huashan Hospital, Fudan University, Shanghai 200040, China.

^2^Department of Infectious Diseases, Huashan Hospital, Fudan University, Shanghai 200040, China

^3^Microbiology Department. Children's Hospital of Fudan University, Shanghai 201102, China

^4^Zhejiang Provincial Demonstration Centre of Laboratory Medicine Experimental Teaching, Wenzhou Medical University, Wenzhou 325035, Zhejiang, China

^5^School of Pharmacy, Fudan University, Shanghai 201203, China

^6^Department of Laboratory Medicine, Jinshan Hospital of Fudan University, Shanghai 201508, China.

†: These authors contributed equally to this work and share first authorship.

* Corresponding authors:

Xiaofei Jiang, PhD, Professor, Department of Laboratory Medicine, Huashan Hospital, Fudan University, Shanghai 200040, China. E-mail address: jiangxi2154@sina.com. Telephone: +86-21-52888316; Fax: +86-21-52888316.

Gang Li, PhD, Department of Laboratory Medicine, Jinshan Hospital of Fudan University, Shanghai 201508, China. E-mail: gorrillee@hotmail.com; Telephone: +86-21-57039466; Fax: +86-21-57039466.

**Supplement**

Table S1 Primers used in this study

| **Primer** | **Sequence (5′-3′)** | **Tm (℃)** | **Product (bp)** |
| --- | --- | --- | --- |
| *rpoB* F | GGCGAAATGGCWGAGAACCA | 61 | 1,076 |
| *rpoB* R | GAGTCTTCGAAGTTGTAACC | 54 |  |
| *gapA* F | TGAAATATGACTCCACTCACGG | 58 | 663 |
| *gapA* R | CTTCAGAAGCGGCTTTGATGGCTT | 64 |  |
| *mdh* F | CCCAACTCGCTTCAGGTTCAG | 61 | 757 |
| *mdh* R | CCGTTTTTCCCCAGCAGCAG | 62 |  |
| *pgi* F | GAGAAAAACCTGCCTGTACTGCTGGC | 66 | 718 |
| *pgi* R | CGCGCCACGCTTTATAGCGGTTAAT | 66 |  |
| *phoE* F | ACCTACCGCAACACCGACTTCTTCGG | 69 | 603 |
| *phoE* R | TGATCAGAACTGGTAGGTGAT | 56 |  |
| *infB* F | CTCGCTGCTGGACTATATTCG | 58 | 463 |
| *infB* R | CGCTTTCAGCTCAAGAACTTC | 58 |  |
| *tonB* F | CTTTATACCTCGGTACATCAGGTT | 58 | 540 |
| *tonB* R | ATTCGCCGGCTGRGCRGAGAG | 65 |  |
| *wzy-K1* F | GGTGCTCTTTACATCATTGC | 55 | 1,283 |
| *wzy-K1* R | GCAATGGCCATTTGCGTTAG | 59 |  |
| *allS* F | CCGAAACATTACGCACCTTT | 57 | 508 |
| *allS* R | ATCACGAAGAGCCAGGTCAC | 60 |  |
| *entB* F | GTCAACTGGGCCTTTGAGCCGTC | 66 | 400 |
| *entB* R | TATGGGCGTAAACGCCGGTGAT | 65 |  |
| *irp2* F | GCTACAATGGGACAGCAACGAC | 62 | 230 |
| *irp2* R | GCAGAGCGATACGGAAAATGC | 60 |  |
| *iroN* F | GTCCGGCGGTAACTTCAGCC | 63 | 829 |
| *iroN* R | TCAGAATGAAACTACCGCCC | 58 |  |
| *iucA* F1 | AATCAATGGCTATTCCCGCTG | 59 | 239 |
| *iucA* R1 | CGCTTCACTTCTTTCACTGACAGG | 62 |  |
| *iucA* F2 | GCTTATTTCTCCCCAACCC | 56 | 583 |
| *iucA* R2 | TCAGCCCTTTAGCGACAAG | 57 |  |
| *fimH* F | TGCTGCTGGGCTGGTCGATG | 65 | 909 |
| *fimH* R | GGGAGGGTGACGGTGACATC | 62 |  |
| *mrkD* F | AAGCTATCGCTGTACTTCCGGCA | 64 | 340 |
| *mrkD* R | GGCGTTGGCGCTCAGATAGG | 64 |  |
| *p-rmpA2* F | GTGCAATAAGGATGTTACATTA | 52 | 230 |
| *p-rmpA2* R | GACTTATCATATTTAATGTT | 43 |  |
| *c-rmpA* F | GTAATAGAGATATAAATATCATATTGA | 49 | 589 |
| *c-rmpA* R | CATCTTTCATCAACCATTTC | 50 |  |
| *p-rmpA* F | GAGTAGTTAATAAATCAATAGCAAT | 52 | 332 |
| *p-rmpA* R | CAGTAGGCATTGCAGCA | 55 |  |
| *peg-344* F1 | CTTGAAACTATCCCTCCAGTC | 55 | 508 |
| *peg-344* R1 | CCAGCGAAAGAATAACCCC | 56 |  |
| *peg-344* F2 | AAAGGACAGAAAGCCAGTG | 55 | 411 |
| *peg-344* R2 | CAATGACGAGGGGGATAATC | 55 |  |
| *wzi* F^1^ | AACGACTTGCGTAACGACCT | 60 | 587 |
| *wzi* R^1^ | TTCATTTGGCTGGCGGAGAT | 60 |  |
| *wzi* F^2^ | GTGCCGCGAGCGCTTTCTATCTTGGTATTC C | 70 | 581 |
| *wzi* R^2^ | GAGAGCCACTGGTTCCAGAACTTCACCGC | 70 |  |
| pKOBEG F | TCCATTACCCGTGCGTTTGA | 60 | 483 |
| pKOBEG R | CGGTAACGCAGATCGGATGA | 60 |  |
| *p-rmpA F* | AAGAGTATTGGTTGACAGCAGGA | 60^4^ | 108 |
| *p-rmpA R* | GAAACGTCAAGCCACATCCA | 60^4^ |  |
| *p-rmpA2 F* | ACGTATGAAGGCTCGATGGAT | 60^4^ | 103 |
| *p-rmpA2 R* | AACCATCCCATTTCCCTGAAT | 60^4^ |  |
| *c-rmpA F* | AGAGTATTGGTTGACTGCAGGA | 60^4^ | 139 |
| *c-rmpA R* | CACACCCTTTAGGGTAAAACCG | 60^4^ |  |
| *manC* F | GTCTCGCGAGCTTTATCCGA | 60^4^ | 132 |
| *manC* R | AAGCGGTGCTCTTCGTTACA | 60^4^ |  |
| *galF* F | GAAGAACGCGGTGGAAAACC | 60^4^ | 131 |
| *galF* R | GCACGTTCATGATGGTCACG | 60^4^ |  |
| *16S rRNA* F | TACCGCATAACGTCGCAAGA | 60^4^ | 149 |
| *16S rRNA* R | TTCCAGTGTGGCTGGTCATC | 60^4^ |  |
| Kan F | ATTACACGTCTTGAGCGATT | 61^3^ | 1497 |
| Kan R | CATATGAATATCCTCCTTAGTTCC | 59^3^ |  |
| *wzi* up F | GAGGTTCGGTGGATAAGAGC | 65^3^ | 530 |
| *wzi* up R | AATCGCTCAAGACGTGTAATCAGCTGACTTCGCTGTGCAA | 68^3^ |  |
| *wzi* down F | GGAACTAAGGAGGATATTCATATGGGCTGAATGCTGAAAACAGG | 64^3^ | 510 |
| *wzi* down R | TCAGAATTAACCCAGTTACCAG | 61^3^ |  |
| *wzy-K1* up F | ATCTGCCACCTGAGTTACAA | 63^3^ | 510 |
| *wzy-K1* up R | AATCGCTCAAGACGTGTAATTGTTTTTCATCCCTTTATGAG | 57^3^ |  |
| *wzy-K1* down F | GGAACTAAGGAGGATATTCATATGTGCAAAAAGATTTAAAATAATAGAGG | 57^3^ | 507 |
| *wzy-K1* down R | TTCGCCATAAGCATTAAGTAAT | 59^3^ |  |
| *cat* F | CCATATGAATATCCTCCTTAGTT | 58^3^ | 1031 |
| *cat* R | GTGTAGGCTGGAGCTGCTTC | 68^3^ |  |
| *wzi* up F | GAGGTTCGGTGGATAAGAGC | 65^3^ | 530 |
| *wzi* up R | AACTAAGGAGGATATTCATATGGCAGCTGACTTCGCTGTGCAA | 68^3^ |  |
| *wzi* down F | GAAGCAGCTCCAGCCTACACGGCTGAATGCTGAAAACAGG | 64^3^ | 510 |
| *wzi* down R | TCAGAATTAACCCAGTTACCAG | 61^3^ |  |

1: regular PCR; 2: regular PCR and sequencing; 3: Q5 High-Fidelity DNA Polymerase condition; 4: qRT-PCR.
